# Supplementary material for: Relationship between working conditions and psychological distress experienced by junior doctors in the UK during the COVID-19 pandemic: a cross-sectional survey study
Source: BMJ Open. 2022 Aug 23;12(8):e061331. doi: 10.1136/bmjopen-2022-061331 (PMC9402444; doi:10.1136/bmjopen-2022-061331)
Supplement: Author's manuscript [file bmjopen-2022-061331.draft_revisions.pdf]

BMJ Open

BMJ Open is committed to open peer review. As part of this commitment we make the peer review history of every article we publish publicly available.

When an article is published we post the peer reviewers' comments and the authors' responses online. We also post the versions of the paper that were used during peer review. These are the versions that the peer review comments apply to.

The versions of the paper that follow are the versions that were submitted during the peer review process. They are not the versions of record or the final published versions. They should not be cited or distributed as the published version of this manuscript.

BMJ Open is an open access journal and the full, final, typeset and author-corrected version of record of the manuscript is available on our site with no access controls, subscription charges or pay-per-view fees (http://bmjopen.bmj.com).

If you have any questions on BMJ Open's open peer review process please email info.bmjopen@bmj.com

BMJ Open

The relationship between working conditions and psychological distress experienced by junior doctors in the UK during the COVID-19 pandemic: A cross-sectional survey study

Journal:	*BMJ Open*
Manuscript ID	bmjopen-2022-061331
Article Type:	Original research
Date Submitted by the Author:	09-Feb-2022

Complete List of Authors:	Dunning, Alice; Bradford Teaching Hospitals NHS Foundation Trust, Research<br>Teoh, Kevin; Birkbeck University of London, Department of Organizational Psychology<br>Martin, James; University of Birmingham, Institute of Applied Health Research<br>Spiers, Johanna; University of Birmingham, College of Medical and Dental Sciences<br>Buszewicz, Marta; University College London, Research Department of Primary Care and Population Health<br>Chew-Graham, Carolyn; Keele University<br>Taylor, Anna; University of Leeds,<br>Gopfert, Anya; Oxford University Hospitals NHS Trust<br>Van Hove, Maria; London School of Hygiene & Tropical Medicine<br>Appleby, Louis; The University of Manchester Faculty of Medical and Human Sciences, Psychiatry & Behavioral Sciences<br>Riley, Ruth; University of Birmingham, MDS;

Keywords:	COVID-19, EDUCATION & TRAINING (see Medical Education & Training), Health & safety < HEALTH SERVICES ADMINISTRATION & MANAGEMENT, Human resource management < HEALTH SERVICES ADMINISTRATION & MANAGEMENT, Organisational development < HEALTH SERVICES ADMINISTRATION & MANAGEMENT, Quality in health care < HEALTH SERVICES ADMINISTRATION & MANAGEMENT

SCHOLARONE™
Manuscripts

I, the Submitting Author has the right to grant and does grant on behalf of all authors of the Work (as defined in the below author licence), an exclusive licence and/or a non-exclusive licence for contributions from authors who are: i) UK Crown employees; ii) where BMJ has agreed a CC-BY licence shall apply, and/or iii) in accordance with the terms applicable for US Federal Government officers or employees acting as part of their official duties; on a worldwide, perpetual, irrevocable, royalty-free basis to BMJ Publishing Group Ltd ("BMJ") its licensees and where the relevant Journal is co-owned by BMJ to the co-owners of the Journal, to publish the Work in this journal and any other BMJ products and to exploit all rights, as set out in our licence.

The Submitting Author accepts and understands that any supply made under these terms is made by BMJ to the Submitting Author unless you are acting as an employee on behalf of your employer or a postgraduate student of an affiliated institution which is paying any applicable article publishing charge ("APC") for Open Access articles. Where the Submitting Author wishes to make the Work available on an Open Access basis (and intends to pay the relevant APC), the terms of reuse of such Open Access shall be governed by a Creative Commons licence – details of these licences and which Creative Commons licence will apply to this Work are set out in our licence referred to above.

Other than as permitted in any relevant BMJ Author's Self Archiving Policies, I confirm this Work has not been accepted for publication elsewhere, is not being considered for publication elsewhere and does not duplicate material already published. I confirm all authors consent to publication of this Work and authorise the granting of this licence.

For peer review only

The relationship between working conditions and psychological distress experienced by junior doctors in the UK during the COVID-19 pandemic: A cross-sectional survey study

Authors: Alice Dunning, Kevin Rui-Han Teoh, James Martin, Johanna Spiers, Marta Buszewicz, Carolyn Chew-Graham, Anna Kathryn Taylor, Anya Gopfert, Maria Van Hove, Louis Appleby, Ruth Riley

Alice Dunning (Corresponding author)

Yorkshire Quality and Safety Research Group

Bradford Institute for Health research

Bradford Teaching Hospitals NHS Foundation Trust

Bradford

United Kingdom

Alice.dunning@bthft.nhs.uk

Kevin Rui-Han Teoh

Department of Organizational Psychology

Birkbeck, University of London

London

United Kingdom

James Martin

Institute of Applied Health Research

University of Birmingham

Birmingham

United Kingdom

Johanna Spiers

Institute of Applied Health Research

University of Birmingham

For peer review only

Birmingham

United Kingdom

Marta Buszewicz

Research Department of Primary Care and Population Health

University College London Medical School

London

United Kingdom

Carolyn A. Chew-Graham

School of Medicine

Keele University

Keele

United Kingdom

Anna K. Taylor

Leeds Institute of Health Sciences

Faculty of Medicine and Health

University of Leeds

Leeds

United Kingdom

Anya Gopfert

Torbay Council,

Torquay,

TQ1 3DR

Maria van Hove

The University of Exeter,

Exeter,

EX4 4PY

For peer review only

Louis Appleby

Division of Psychology and Mental Health

School of Medicine

University of Manchester

Manchester

United Kingdom

Ruth Riley

Institute of Applied Health Research

University of Birmingham

Birmingham

United Kingdom

For peer review only

Abstract

Objectives: This paper explored the self-reported prevalence of depression, anxiety and stress among junior doctors during the COVID-19 pandemic. It also reports the association between working conditions and psychological distress experienced by junior doctors.

Design: A cross-sectional online survey study was conducted, utilising the DASS-21 and HSE scale to measure psychological wellbeing and working cultures of junior doctors.

Setting: The National Health Service in the UK.

Participants: A sample of 456 UK junior doctors was recruited online during the COVID-19 pandemic from March 2020-January 2021.

Results: Junior doctors reported poor mental health, with over 40% scoring extremely severely depressed (45.2%), anxious (63.2%) and stressed (40.2%). Both gender and ethnicity were found to have a significant influence on levels of anxiety. Hierarchical multiple linear regression analysis outlined the specific working conditions which significantly predicted depression (increased

demands, relationships, unsupportive manager), anxiety (relationships, change, demands) and stress (relationships, demands, role).

Conclusions: The findings illustrate the importance of working conditions for junior doctors' mental health, as they were significant predictors for depression, anxiety and stress. Therefore, if the mental health of junior doctors is to be improved, it is important that changes or interventions specifically target the working environment rather than factors within the individual clinician.

Article summary: Strengths and limitations

- The study outlines the association between working conditions and psychological distress for junior doctors which has important implications for future interventions, suggesting the need for them to focus upon the environment rather than individual.
- This is the first study to utilise the Health Standards Executive measure within a National Health Service setting to explore the working conditions of junior doctors.
- The sample of participants showed diverse representation across ethnicity, specialties and years of experience. The findings outline differences reported between genders and ethnicities.
- However, the sample was not proportionately representative, as the majority of participants were white and female.

Background

The demands of workload and job expectations on healthcare workers have been widely acknowledged,[1]. These demands have been further exacerbated by the COVID-19 pandemic, which created unprecedented working conditions for these workers,[2]. Prior to the pandemic the 2019 National Health Service (NHS) staff survey found that 40% staff reported feeling unwell due to work-related stress in 5 years,[3]. The 2019 NHS Long Term Plan,[4] outlined the impact of increased demands on healthcare staff wellbeing. Ten years previously, the NHS Health and Well-Being report

For peer review only

argued that staff wellbeing should be at the centre of all organisations in the NHS,[5]. However, despite acknowledgment of the demanding work environments and poor wellbeing among healthcare workers in the NHS, relatively little has been done to better support staff,[6].

Doctors report more anxiety and depression than the general population,[7]. A particularly vulnerable group of doctors to experiencing distress are junior doctors. Junior doctors are qualified doctors in training or doctors who are not consultants; this title also includes foundation year doctors,[8] (BMA, 2020). The General Medical Council (GMC) has highlighted the low morale, distress and alienation experienced by junior doctors,[9] (GMC, 2016). Junior doctors and medical students reported the highest rates of being diagnosed with a mental illness, including anxiety and depression during the previous 12 months compared to others doctors; of these, 91% of junior doctors were at high or very high risk of burnout,[10]. Furthermore, Irish junior doctors reported significantly higher levels of psychological distress in comparison to consultants,[11]. Junior doctors' have also reported feeling dehumanised by their employers,[12]. Evidence suggests that junior doctors are more vulnerable to feeling distressed which are attributed to feelings of being unsupported, devalued, without autonomy,[13] (Health Education England, 2017) A barrier for junior doctors seeking help for mental health concerns is the fear of failing if off sick,[14](Fox et al., 2011).

Research has identified gender differences in the mental health of UK doctors. One survey found that females showed a higher risk of burnout and were 50% more likely to report a mental illness diagnosis compared to their male counterparts,[10]. Although there are mixed findings about suicide rates internationally, there are concerns over suicidality among doctors, particularly amongst female doctors,[15] (ONS, 2017).

Frontline healthcare professionals have reported increased levels of distress whilst working during the COVID-19 pandemic,[16]. Furthermore, research has demonstrated the increased risk of burnout for NHS doctors at this time, with nearly half describing deterioration of their mental health,[17] (Cubitt et al., 2021). High levels of psychological distress have been reported among anaesthesiology, emergency medicine and intensive care doctors in both Ireland and the UK,[18].

For peer review only

The increased work demands, redeployment, inadequate access to basic facilities or safe spaces to rest, loss of autonomy and anxiety around the plans for recovery were identified as contributing factors to the deterioration of their mental health,[17, 19]. The pandemic has also had a significant impact on training and further exacerbated the levels of poor mental health among junior doctors, with 70% experiencing anxiety, depression and burnout,[20].

Working conditions as a predictor of junior doctors' mental health

In light of the prevalence of poor mental health among junior doctors, it is important to understand the relevant antecedent factors. While workplace health interventions typically focus on addressing individual-level factors in the behaviour or approach of junior doctors (e.g., coping behaviours and resilience), there is increasing recognition that how workplaces are organised, designed and managed are more important contributing factors, not only to doctors' mental health but also towards patient care,[21, 22]. Two work-related factors, including high demands and a poor environment, were identified as being associated with higher levels of burnout among junior doctors within a systematic review of 48 studies,[23]. An Australian survey found that increasing working hours led to an increase in mental health problems among junior doctors,[7]. Qualitative research in Australia identified support from colleagues as improving junior doctors' levels of burnout and wellbeing,[24] (Hoffman, 2018). Similarly, interviews with junior doctors in the UK identified poor working conditions, toxic work cultures, lack of support and mental health stigma as being contributing factors to poor levels of wellbeing among junior doctors,[25].

To better, and more holistically, support the mental health of junior doctors it is important to understand the specific working conditions within the UK health system related to their mental health. Although other studies have correlated other measures of work environment with the mental health of NHS doctors (e.g.,[22]) or nurses,[26, 27], this has not been done specifically for junior doctors.

Aims

For peer review only

To explore the prevalence of depression, anxiety and stress experienced by junior doctors in the UK during the COVID-19 pandemic.

To identify working conditions associated with psychological distress.

Method

Study design and sample

A cross-sectional online survey, conducted as part of a wider mixed methods study,[25, 28], was utilised to measure the working conditions and mental health of a convenience sample of UK junior doctors. Participants were recruited online from March 2020 until January 2021 through advertisements on social media posts, junior doctor forums and emails circulated via specialist schools. The survey was hosted on the online platform Qualtrics, which allowed participants to access the questionnaire at a time which suited them. Participants were entered into a prize draw for shopping vouchers (three prizes of £50 were available) to thank them for their time.

Measures

Demographic information

Age, gender, ethnicity, sexuality, years as a doctor and speciality were recorded at the beginning of the online survey.

Mental Health

Levels of depression, anxiety and stress were all measured using the 21-item Depression, Anxiety and Stress Scale (DASS-21),[29] (Lovibond & Lovibond, 1995). Participants rated the extent to which they had experienced each item within the past week on a four-point Likert scale. Following the scale guidelines, participant scores for depression, anxiety and stress were multiplied by two, and categorised into normal, mild, moderate, severe and extremely severe, following the recommended cut-off scores.

Working conditions

The UK Health and Safety Executive's (HSE) management standards framework was utilised. This identifies seven key aspects of the working environment using a 35-item scale: role clarity; peer

For peer review only

support; strained relationships; managerial support; work demands; control over working environment; and change. These are based on relevant occupational health psychology theories (e.g., the job demands resources model),[30] (Bakker & Demerouti, 2017). While the framework has been widely applied across different sectors (e.g.,[31-33]), we are not aware of its application in the NHS. Participants were asked to rate how often they experienced each item within the last six months on a five-point Likert scale.

Ethical considerations

Participants were given an information sheet and asked to provide online consent prior to responding to the survey. All identifiable information was stored securely and separately to non-identifiable survey data. The research was approved by the University of Birmingham ethics committee and Health Research Authority (reference number: 19/HRA/6579)

Patient and Public Involvement

No patient involvement.

Data Analysis

After data was extracted from Qualtrics, we carried out data management and analysis using SPSS version 26. Measures were scored according to their instructions and, where appropriate, items reverse scored so that a higher score represents a stronger measure of the construct. Participant demographics were summarised using numbers and percentages, means and standard deviations or median and inter-quartile ranges, as appropriate. A Shapiro-Wilk test was used to assess normality. The normality of the variables was not established as all the measures were skewed and had a kurtosis score greater than the absolute value of 2.0. Little's chi-square statistic for testing whether values are missing completely at random (MCAR),[34] (Little, 1988) was not significant (X^2 (1746, 456) = 1145.09, p=1.00). This demonstrates that there was no pattern to the missing values within the data set. To test for association between participants characteristics and the depression, anxiety, and stress scores, we used Kruskall-Wallis non-parametric tests with

For peer review only

Bonferonni adjustments to test against the raw scores, and chi-squared tests to test against the classified scores. We then carried out Spearman's Rho correlations between the study variables as assumptions of normality were not met.

To quantify the effect of working conditions on each of the outcomes (depression, anxiety, and stress), we fitted two linear regression models to each outcome. Our first model included only the participants' demographics (age, gender, years as a doctor). The second model included participants' demographics and the seven working conditions (demands, control, manager support, peer support, role, change, strained relationships).

Results

Participant characteristics and levels of depression, anxiety and stress

A total of 456 responses were received with the mean age of 30.70 (*SD* = 4.87). The mean number of years working as a doctor was 4.94 (*SD* = 3.56). Most participants identified as female (67.1%) and came from a white background (71.1%), 80% of the sample identified as heterosexual. A full demographic breakdown is presented in Table 1.

[Insert Table 1 here]

Table 1 provides an overview of the proportion of the sample that were categorised on each degree of the mental health measures. Across all three measures, at least 70% of the sample scored 'severe' or 'extremely severe'. For depression, 45.2% of participants were classed as having extremely severe levels of depression, with an additional 23.3% classed as severe (p<.001). There were more participants classed as having extremely severe levels of anxiety (63.2%) compared to those with severe (25.2%) or moderate (11.7%) levels (p<0.001). No participants had normal or mild levels of depression or anxiety. More participants also reported extremely severe (40.2%) or severe (31.5%) levels of stress than less severe levels (p<.001). Chi-square analysis between participant

For peer review only

characteristics (i.e., gender, ethnicity, sexeuality) and classification of depression, anxiety and stress showed no significant associations (Table 1).

Table 2: Mean comparisons between participants' characteristics for levels of depression, anxiety and stress.

	Depression			Anxiety			Stress		
Characteristic	Mean	*S.D.*	*p* value	Mean	*S.D.*	*p* value	Mean	*S.D.*	*p* value
Gender	28.21	11.30	.44	24.94	10.32	.03	30.97	9.60	.29
Male	28.91	11.47		23.71	10.58		30.37	10.25	
Female	27.92	11.23		25.45	10.18		31.21	9.32	
Ethnicity	28.50	11.47	.01	25.15	10.41	.01	31.14	9.63	.77
White	27.74	27.80		24.16	9.77		30.81	9.45	
Asian	31.51	12.84		27.88	11.42		31.82	10.08	
Black	26.75	10.79		32.50	13.17		31.00	11.76	
Mixed	24.80	9.48		25.04	20.23		30.72	8.18	
Other	34.76	12.70		28.00	12.79		34.19	11.52	
Sexuality	28.51	11.52	.10	25.12	10.43	.26	31.10	9.64	.66
Heterosexual	28.17	11.37		24.76	10.24		30.90	9.73	
LGBTQ+	28.83	11.88		26.21	10.93		32.12	9.15	
Prefer not to say	34.93	12.23		28.67	12.32		31.20	9.97	

Note. *p* values are for differences between means within each characteristic

We found no evidence of differences on levels of depression (p=.439) and stress (p=.290) between male and female participants (Table 3). However, significant differences were reported for anxiety (p=.032), were participants who identified as female (M = 25.45) reported a higher mean score for anxiety than male (M = 23.71). No differences were observed based on their sexual orientation for levels of depression (p=.098), anxiety (p=.260), or stress (p=.662).

For peer review only

In terms of ethnicities, a Kruskall-Wallis (Table 2) found a significant difference in levels of depression (*p*=.016). Post-hoc comparison using Bonferroni test showed participants from mixed (*M* = 24.80) background reporting lower levels of depression than those from an Asian (*M* = 31.51) or White (*M* = 27.74) background. Participants from a White (*M* = 27.74) background also reported lower levels of a depression than those from an Other (*M* = 34.76) and from an Asian (*M* = 31.55) background. Differences were also observed across ethnicities for anxiety (*p*=.020), with Bonferroni comparisons showing that participants from an Asian background (*M* = 27.88) reported higher levels of anxiety than those from a white background (*M* = 24.16). No differences were observed for stress (*p* = .770) between participants from different ethnic groups.

Descriptive statistics and relationships for perceived working conditions, depression, anxiety, and stress

Table 3 presents the descriptive statistics and internal reliabilities for all variables in this study, and that all composite measures had adequate internal consistency (α >.70). Bivariate two-tailed non-parametric correlations show the presence of all anticipated relationships in the appropriate directions. More specifically, reporting more challenging working conditions (i.e., high demands, strained relationships) were was associated with lower levels of positive working conditions (i.e., control, manager support, peer support, role, change), as well as higher levels of depression, anxiety and stress. Higher levels of the four positive working conditions were correlated with lower levels of junior doctors' levels of depression, anxiety and stress.

[Insert Table 3 here]

Direct effects between perceived working conditions and levels of depression, anxiety and stress

Table 4 presents report that junior doctors' strained relationships (β = .27) positively predicted levels of depression. Having good peer support was negatively associated with levels of

For peer review only

depression (β = -.15). There was no evidence of an association between participants' characteristics (age, gender, and years as a doctor) and depression.

Table 4: Multiple linear regression analyses with depression, anxiety and stress as dependant variables

	Step 1		Step 2	
Predictors	β	B [95% CI]	β	B [95% CI]
Depression				
Age	.048	0.110 (-0.216, 0.435)	.007	0.017 (-0.259, 0.292)
Gender (Female)	-.027	0.675 (-3.156, 1.807)	-.012	-0.305 (-2.400, 1.791)
Years as doctor	-.036	-0.110 (-.0.543, 0.324)	.004	0.014 (-0.359, 0.386)
Demands			.101	1.683 (-0.187, 3.552)
Control			-.017	-.273 (-2.245, 1.699)
Manager support			-.111	-1.323 (-3.066, 0.420)
Peer			-.147	-2.266 (-4.251, -0.281)
Strained relationships			.273	3.954 (2.314, 5.593)
Role			-.088	-1.699 (-3.775, 0.377)
Change			.039	.550 (-1.107, 2.208)
R^2		0.002		0.324
ΔR^2		0.002		0.322
p value		0.845		0.001
Anxiety				
Gender (Female)	0.079	1.784 (-0.443, 4.000)	.108	2.457 (0.458, 4.457)
Years as doctor	-0.095	-0.266 (0.653, 0.122)	-.083	-.233 (-0.589, 0.122)
Age	-0.088	-0.184 (-0.475, 0.106)	-.120	-.251 (-0.514, 0.012)
Demands			.179	2.716 (0.932, 4.500)
Control			.028	.413 (-1.469, 2.295)
Manager support			.009	.100 (-1.563, 1.763)
Peer			-.105	-1.476 (-3.369, 0.418)
Strained relationships			.314	4.135 (2.571, 5.699)
Role			-.051	-.886 (-2.867, 1.095)
Change			.104	1.328 (-0.3254, 2.909)
R^2		0.037		0.256
ΔR^2		0.037		0.219

For peer review only

p value		0.002		0.001
Stress				
Gender (Female)	.049	1.029 (-1.081, 3.140)	.061	1.298 (-0.511, 3.108)
Years as doctor	-.034	-0.09 (-0.459, 0.279)	.022	.057 (-0.266, 0.379)
Age	.019	0.038 (-0.239, 0.314)	-.027	-.054 (-0.291, 0.184)
Demands			.294	4.164 (2.549, 5.479)
Control			.065	.879 (-0.824, 2.582)
Manager support			-.034	-.346 (-1.851, 1.160)
Peer			-.103	-1.352 (-3.067, 0.363)
Strained relationships			.181	2.225 (0.807, 3.644)
Role			-.119	-1.943 (-3.735, -0.151)
Change			-.007	-.089 (-1.521, 1.343)
R^2		0.003		0.304
ΔR^2		0.003		0.301
p value		0.764		0.001

Note. * *p* < .05, ** *p* < .01; CI: 95% unstandardised confidence intervals.

For anxiety, junior doctors who identified as female reported higher levels of anxiety (β = .11) than those who identified as male. Table 4 shows that strained relationships were the strongest predictor (β = .31) of anxiety, followed by how well change is managed (β = .18.

Focusing on stress, we see that demographic factors made no contribution to the explained variance in levels of stress. Strained relationships (β = .18) and demands (β = .28) positively predicted stress. In addition, role clarity was associated with lower levels of stress (β = .11).

Discussion

The findings show that junior doctor survey respondents reported high levels of depression, anxiety, and stress during the pandemic, with at least 70% reporting 'severe' or 'extremely severe' across each measure. This study expands what has already been described in the literature by showing that specific working conditions are associated with junior doctors' levels of psychological distress, namely depression (strained relationships, managerial support), anxiety (strained

For peer review only

relationships, organisational change, high demands) and stress (strained relationships, high demands and role).

The high levels of depression anxiety and stress which were reported by junior doctors in this study build upon previous literature which reports elevated psychological distress among junior doctors as compared to pre-pandemic levels,[11]. Crucially, these figures are higher than pre-pandemic comparisons of samples including doctors in the NHS,[35] and Ireland,[11] reflecting the increased strain which healthcare staff experienced whilst working on the frontline,[2, 17, 20]

Furthermore, female junior doctors in this study reported higher levels of anxiety compared with their male counterparts. As increased levels of anxiety are associated with suicidality,[36] this link may provide some insight into the elevated risk of suicide among female doctors,[11]. This difference in anxiety between females and males may be explained by work-related factors such as poorer work life balance,[12]. Further research should explore levels of suicidality among junior doctors as well as potential gender differences. There were also significant differences between levels of anxiety experienced by different ethnicities within this study, with junior doctors from an Asian background reporting higher levels than junior doctors from a White background. While no significant differences were observed for other ethnic groups, ethnic minorities in almost all instances reported poorer levels of mental health. It may be that the lack of statistical power led to the lack of more significant differences being found. This finding is supported by previous literature, which outlines the increased risk of mental health problems for ethnic minorities,[37]. This study is the first to demonstrate an elevated risk of poor mental health among junior doctors an Asian background. This difference between ethnicities may be explained by poorer working conditions which are experienced by ethnic minorities ,[38] or the increased likelihood of experiencing harassment in the workplace,[25, 39]. Future research should explore whether there are additional factors associated with differences between gender, ethnicity, and anxiety; such data could support targeted changes or interventions to support any at risk group.

For peer review only

Working conditions as an antecedent to junior doctors' mental ill-health

The most consistent predictor of depression, anxiety and stress was strained relationships. Previous literature supports this finding, as bullying or witnessing harassment has been found to impact NHS healthcare staff wellbeing and job satisfaction,[40]. Furthermore, toxic working cultures, including bullying, discrimination and a blame culture, were found to be the most frequently discussed source of distress for junior doctors,[25]. A lack of supportive colleagues during incidents or through discontinuity in teams was also highlighted by Riley and colleagues,[28] as a contributing factor to psychological distress in junior doctors.

Additionally, an online survey found the most reported factor for boosting junior doctor morale in the UK was feeling part of a team,[41] demonstrating the impact positive relationships can have. Further, belonging, feeling valued and connected with colleagues and supervisors/consultants/mangers is protective and is associated with better morale and positive mental health among staff,[42]. In line with these findings, interventions which centre on creating supportive working relationships, such as peer mentoring programmes, have been found to have a positive effect on psychological wellbeing and job satisfaction for junior doctors,[43].

Belonging, feeling valued and connectedness are protective factors and can promote positive mental health and a positive professional identity; for instance, peer/colleague support in the workplace has been found to mitigate the negative psychological consequences of adverse events. In line with the evidence that supportive relationships with colleagues are important for mitigating psychological distress, supportive managers were found to be significantly associated with lowerlevels of depression in this study. The importance of supportive leadership for healthcare staff,[42] and doctors',[22] wellbeing has been previously identified. Supportive relationships and feeling valued by supervisors/consultants acts a buffer against the demands of the job,[28]. Taken together, the findings that strained relationships and supportive leadership are associated with mental health demonstrate the need for a supportive environment, in which junior doctors' work within consistent teams under good leadership to be adopted.

For peer review only

Intense work demands was also associated with psychological distress, demonstrating its importance for junior doctor wellbeing. Previous literature has outlined the increasing strain on healthcare workers because of the demands of their role,[1] which were exacerbated through the pandemic,[2, 17]. Considering the demands healthcare staff face, the prevalence of poor mental health found in this study is unsurprising. This association between high/intense job demands and poor mental health is supported by previous research where job demands predicted doctors' reported wellbeing ,[43](Teoh, Hassard & Cox, 2021), were associated with junior doctors' levels of burnout,[23] and contributed to junior doctors' psychological distress,[25]. This research further highlights the need to address the demanding working conditions which are impacting on junior doctors' distress.

A key strength of this study is the diverse representation across ethnicities, different specialities and years of experience. However, most of the junior doctors in this study were white females. Considering the gender and ethnicity differences reported in the results, a more proportionally representative sample would have improved the generalisability of the results. Nonetheless, the findings from this study provide a valuable understanding of the relationship between working conditions and mental health for junior doctors.

Conclusion

In summary, the findings of this study reflect the poor levels of psychological wellbeing many junior doctors' experienced during the pandemic. Our results demonstrate the association between working conditions, specifically job demands, strained relationships, organisational change and role certainty and the mental health of junior doctors. This indicates the need for systemic changes or interventions that focus on improving the working conditions of junior doctors rather than focusing on 'resilience' in the individual.

Author Contribution: All authors contributed to conception, design, data collection, analysis, drafting and approval of final version of the article to be published. AD acts as guarantor for this paper.

For peer review only

Acknowledgements: The authors would like to thank all junior doctor respondents who kindly gave their time to complete the survey. We also wish to thank members of the PPIE group who provided valuable input throughout the study.

Competing interests: None

Funding: The study was funded by NIHR Research for Patient Benefit (PB-PG-0418-20023)

Department of Health Disclaimer: The views and opinions expressed therein are those of the authors and do not necessarily reflect those of the NIHR, NHS or the Department of Health.

Ethical approval: Ethical approval was granted by the University of Birmingham and Health Research Authority (reference number: 19/HRA/6579)

Data sharing statement: This study has not received ethical approval to share confidential data with any third party other than the study research team.

For peer review only

Table 1. Frequency and percentages of participant demographics mapped against degree of depression, anxiety, and stress.

	N	Depression					Anxiety					Stress					
		Normal / Mild	Moderate	Severe	Extremely Severe	*p* value	Normal/ Mild	Moderate	Severe	Extremely Severe	*p* value	Normal	Mild	Moderate	Severe	Extremely severe	*p* value
Total	456 (100%)	0 (0%)	135 (31.4%)	100 (23.3%)	194 (45.2%)	.01*	0 (0%)	50 (11.7%)	108 (25.2%)	271 (63.2%)	.01*	8 (1.9%)	39 (9.1%)	74 (17.3%)	135 (31.5%)	172 (40.2%)	.01*
Gender						.78					.22						.67
Male	123 (27%)	-	33 (28.7%)	30 (26.1%)	52 (45.2%)		-	16 (13.9%)	35 (30.4%)	64 (55.7%)		3 (2.6%)	11 (9.6%)	25 (21.7%)	33 (28.7%)	43 (37.4%)	
Female	306 (67.1%)	-	96 (33.1%)	65 (22.4%)	129 (44.5%)		-	31 (10.7%)	72 (24.8%)	187 (64.5%)		4 (1.4%)	28 (9.7%)	45 (15.6%)	97 (33.6%)	115 (39.8%)	
Prefer to self-describe	4 (0.9%)	-	1 (33.9%)	1 (33.9%)	1 (33.9%)		-	-	-	3 (100%)		-	-	1 (33.9%)	1 (33.9%)	1 (33.9%)	
Prefer not to say	23 (5%)	-	1 (14.3%)	1 (14.3%)	5 (71.4%)		-	2 (28.6%)	-	5 (71.4%)		-	-	2 (28.6%)	-	5 (71.4%)	
Ethnicity						.28					.07						.93
White	324 (71.1%)	-	100 (32.8%)	72 (23.6%)	133 (43.6%)		-	39 (12.8%)	83 (27.2%)	183 (60%)		7 (2.3%)	27 (8.9%)	53 (17.4%)	99 (32.6%)	118 (38.8%)	
Mixed	26 (6/4%)	-	10 (38.5%)	8 (30.8%)	8 (30.8%)		-	2 (7.7%)	9 (34.6%)	15 (57.7%)		-	3 (11.5%)	3 (11.5%)	10 (38.5%)	10 (38.5%)	
Asian	72 (15.8%)	-	18 (26.1%)	16 (23.2%)	35 (50.7%)		-	4 (5.8%)	11 (15.9%)	54 (78.3%)		1 (12.5%)	7 (10.1%)	10 (14.5%)	21 (30.4%)	30 (43.5%)	
Black	9 (2%)	-	3 (37.5%)	2 (25%)	3 (37.5%)		-	-	2 (25%)	6 (75%)		-	1 (12.5%)	2 (25%)	2 (25%)	3 (37.5%)	
Other	25 (5.5%)	-	4 (19%)	2 (9.5%)	15 (71.4%)		-	5 (23.8%)	3 (14.3%)	13 (61.9%)		-	1 (4.8%)	6 (28.6%)	3 (14.3%)	11 (52.4%)	
Sexuality						.42					.49						.17
Heterosexual	365	-	113	79	152		-	43	89	212		8	33	57	107	138	

For peer review only

	(80%)		(32.8%)	(23%)	(44.2%)		(12.5%)	(25.9%)	(61.6%)	(2.3%)	(84.6%)	(31.2%)	(31.2%)	(40.2%)
LGBTQ+	69 (15.2%)	-	18 (27.7%)	18 (27.7%)	29 (44.6%)	-	4 (6.2%)	17 (26.2%)	44 (67.7%)	-	5 (7.7%)	10 (15.4%)	26 (40%)	24 (36.9%)
Prefer not to say	21 (4.6%)	-	3 (20%)	2 (13.3%)	10 (66.7%)	-	2 (13.3%)	2 (13.3%)	11 (73.3%)	-	1 (6.7%)	6 (40%)	1 (6.7%)	7 (46.7%)

For peer review only

Table 3: Descriptive statistics, correlations, and reliability coefficients among study variables

Variable	Internal consistency (Cronbach α)	1	2	3	4	5	6	7	8	9	10	11	12
1. Years as doctor	-	4.94 (3.56)	.70**	-.09*	.15**	.10*	.04	.04	.20**	.09	-.02	-.17**	-.03
2. Age	-		30.70 (4.87)	-.04	.10*	.04	-.06	.07	.06	-.01	.03	-.16**	-.04
3. Demands	.86			3.19 (0.68)	-.60**	-.52**	-.45**	.53**	-.42**	-.43**	.40**	.34**	.45**
4. Control	.84				2.65 (0.71)	.66**	.52**	-.42**	.43**	.58**	-.37**	-.29**	-.34**
5. Manager support	.90					2.98 (0.95)	.71**	-.54**	.51**	.66**	-.46**	-.30**	-.39**
6. Peer support	.84						3.61 (0.73)	-.56**	.48**	.46**	-.45**	-.32**	-.38**
7. Strained Relationships	.84							2.34 (0.)	-.45**	-.41**	.49**	.36**	.42**
8. Role	.74								3.80 (0.58)	.46**	-.36**	-.25**	-.35**
9. Change	.80									2.42 (0.81)	-.37**	-.20**	-.33**
10. Depression	.93										28.50 (11.47)	.56**	.72**
11. Anxiety	.79											25.15 (10.41)	.70**
12. Stress	.88												31.14 (9.63)

Note. Mean (*SD*) are reported diagonally; ***p*<.01 level (2-tailed). **p*<.05 level (2-tailed).

References

1 Lacobucci, G. (2015). Staff burnout threatens plans for NHS, says think tank. *BMJ: British Medical Journal (Online)*, *350*.

2 Greenberg, N., & Tracy, D. (2020). What healthcare leaders need to do to protect the psychological well-being of frontline staff in the COVID-19 pandemic. *BMJ Leader*.

3 NHS Staff Survey (2017). National Briefing Published March 2018. NHS Survey Coordination Centre.

4 Department of Health (2019), The NHS Long Term Plan, Available at: https://www.longtermplan.nhs.uk/wp-content/uploads/2019/08/nhs-long-term-planversion-1.2.pdf [accessed August 2020]

5 Boorman S (2009), NHS Health and Well-being Review: Interim Report: Available at: https://webarchive.nationalarchives.gov.uk/20130103004910/http://www.dh.gov.uk/en /Publicationsandstatistics/Publications/PublicationsPolicyAndGuidance/DH_108799 [accessed August 2020]

6 Bajorek, Z., & Holmes, J. (2020). Health and Wellbeing Interventions in Healthcare.

7 Petrie, K., Crawford, J., LaMontagne, A. D., Milner, A., Dean, J., Veness, B. G., ... & Harvey, S. B. (2020). Working hours, common mental disorder and suicidal ideation among junior doctors in Australia: a cross-sectional survey. *BMJ open*, *10*(1), e033525.

10 Bhugra, D., Sauerteig, S. O., Bland, D., Lloyd-Kendall, A., Wijesuriya, J., Singh, G., ... & Ventriglio, A. (2019). A descriptive study of mental health and wellbeing of doctors and medical students in the UK. *International Review of Psychiatry*, *31*(7-8), 563-568.

11 Hayes, B., et al., *What's up doc? A national cross-sectional study of psychological wellbeing of hospital doctors in Ireland.* BMJ Open, 2017. **7**(10).

12 Rich, A., Viney, R., Needleman, S., Griffin, A., & Woolf, K. (2016). 'You can't be a person and a doctor': the work–life balance of doctors in training—a qualitative study. *BMJ open*, *6*(12), e013897.

13 NHS., *Junior doctor morale. Understanding best practice working environments*. 2017, Health Education England.

14 Fox, F. E., Doran, N. J., Rodham, K. J., Taylor, G. J., Harris, M. F., & O'Connor, M. (2011). Junior doctors' experiences of personal illness: a qualitative study. *Medical education*, *45*(12), 1251-1261.

15 Office for National Statistics, 2017. Suicides in Great Britain: 2016 Registrations. https://www.ons.gov.uk/releases/suicidesingreatbritain2016 [accessed August 2020]

16 Greenberg, N., Weston, D., Hall, C., Caulfield, T., Williamson, V., & Fong, K. (2021). Mental health of staff working in intensive care during COVID-19. *Occupational Medicine*, *71*(2), 62-67.

17 Cubitt, L. J., Im, Y. R., Scott, C. J., Jeynes, L. C., & Molyneux, P. D. (2021). Beyond PPE: a mixed qualitative–quantitative study capturing the wider issues affecting doctors' well-being during the COVID-19 pandemic. *BMJ open*, *11*(3), e050223.

18 Roberts, T., Daniels, J., Hulme, W., Hirst, R., Horner, D., Lyttle, M. D., ... & Carlton, E. (2021). Psychological distress during the acceleration phase of the COVID-19 pandemic: a survey of doctors practising in emergency medicine, anaesthesia and intensive care medicine in the UK and Ireland. *Emergency Medicine Journal*, *38*(6), 450-459

19 Spiers, J., Buszewicz, M., Chew-Graham, C., Dunning, A., Taylor, A. K., Gopfert, A., ... & Riley, R. (2021). What challenges did junior doctors face while working during the COVID-19 pandemic? A qualitative study. *BMJ open*, *11*(12), e056122.

20 Dekker, A., Lavender, D., Clarke, D., & Tambe, A. (2020). How has the COVID-19 pandemic affected junior doctor training? A survey analysis. *The Transient Journal of Trauma, Orthopaedics and the Coronavirus*.

For peer review only

21 De Lange, A. H., Løvseth, L. T., Teoh, K. R. H., & Christensen, M. (2020). Healthy healthcare: empirical occupational health research and evidence-based practice. *Frontiers in Psychology*, *11*, 2236.

22 Teoh, K. R. H., Hassard, J., & Cox, T. (2021). Doctors' working conditions, wellbeing and hospital quality of care: A multilevel analysis. *Safety Science*, *135*, 105115.

23 Zhou, A. Y., Panagioti, M., Esmail, A., Agius, R., Van Tongeren, M., & Bower, P. (2020). Factors associated with burnout and stress in trainee physicians: a systematic review and meta-analysis. *JAMA network open*, *3*(8), e2013761-e2013761.

24 Hoffman, R., & Bonney, A. (2018). Junior doctors, burnout and wellbeing: Understanding the experience of burnout in general practice registrars and hospital equivalents. *Australian journal of general practice*, *47*(8), 571-575.

25 Riley, R., Buszewicz, M., Kokab, F., Teoh, K., Gopfert, A., Taylor, A. K., ... & Chew-Graham, C. (2021). Sources of work-related psychological distress experienced by UK-wide foundation and junior doctors: a qualitative study. *BMJ open*, *11*(6), e043521.

26 Brunetto Y, Xerri M, Trinchero E, et al. Comparing the impact of management on public and private sector nurses in the UK, Italy, and Australia. Public Manag Rev. 2018;20(4):525-544.

27 Yoshida Y, Sandall J. Occupational burnout and work factors in community and hospital midwives: A survey analysis. Midwifery. 2013;29(8):921-926.

28 Riley, R., Kokab, F., Buszewicz, M., Gopfert, A., Van Hove, M., Taylor, A. K., ... & Chew-Graham, C. (2021). Protective factors and sources of support in the workplace as experienced by UK foundation and junior doctors: a qualitative study. *BMJ open*, *11*(6), e045588.

29 Lovibond, S. H., & Lovibond, P. F. (1996). *Manual for the depression anxiety stress scales*. Psychology Foundation of Australia.

For peer review only

30 Bakker, A. B., & Demerouti, E. (2017). Job demands–resources theory: taking stock and looking forward. *Journal of occupational health psychology*, *22*(3), 273.

31 Houdmont, J., Kerr, R., & Randall, R. (2012). Organisational psychosocial hazard exposures in UK policing: Management Standards Indicator Tool reference values. *Policing: An International Journal of Police Strategies & Management*.

32 Kinman, G., Clements, A. J., & Hart, J. (2017). Job demands, resources and mental health in UK prison officers. *Occupational Medicine*, *67*(6), 456-460.

33 Wattie, S. C., & Bridger, R. S. (2019). Work-related stress indicator surveys in UK Ministry of Defence. *BMJ Military Health*, *165*(2), 128-132.

34 Little, R. J. (1988). A test of missing completely at random for multivariate data with missing values. *Journal of the American statistical Association*, *83*(404), 1198-1202.

35 Cramer, R. J., Ireland, J. L., Hartley, V., Long, M. M., Ireland, C. A., & Wilkins, T. (2020). Coping, mental health, and subjective well-being among mental health staff working in secure forensic psychiatric settings: Results from a workplace health assessment. *Psychological services*, *17*(2), 160.

36 Thibodeau, M. A., Welch, P. G., Sareen, J., & Asmundson, G. J. (2013). Anxiety disorders are independently associated with suicide ideation and attempts: propensity score matching in two epidemiological samples. *Depression and anxiety*, *30*(10), 947-954.

37Maura, J., & de Mamani, A. W. (2017). Mental health disparities, treatment engagement, and attrition among racial/ethnic minorities with severe mental illness: A review. *Journal of Clinical Psychology in Medical Settings*, *24*(3), 187-210.

38 NHS., *Workforce Race Equality Standard report*. 2021, NHS England

For peer review only

39 Rhead, R. D., Chui, Z., Bakolis, I., Gazard, B., Harwood, H., MacCrimmon, S., ... & Hatch, S. L. (2021). Impact of workplace discrimination and harassment among National Health Service staff working in London trusts: results from the TIDES study. *BJPsych open*, *7*(1).)

40 Carter M, Thompson N, Crampton P, et al. Workplace bullying in the UK NHS: a questionnaire and interview study on prevalence, impact and barriers to reporting. BMJ Open 2013;3:e002628

41 Singh, R., Kirtley, J., Minhas, J. S., Lakhani, D., & Carr, S. (2019). Exploring junior doctor morale in a UK hospital. *The journal of the Royal College of Physicians of Edinburgh*, *49*(4), 312-316.

42 Borrill C, West M, Shapiro D, et al. Team working and effectiveness in health care. British Journal of Healthcare Management 2000;6:364–71

43 Teoh, K. R. H., Hassard, J., & Cox, T. (2021). Doctors' working conditions, wellbeing and hospital quality of care: A multilevel analysis. *Safety Science*, *135*, 105115.

For peer review only

Reporting checklist for cross sectional study.

Based on the STROBE cross sectional guidelines.

Instructions to authors

Complete this checklist by entering the page numbers from your manuscript where readers will find each of the items listed below.

Your article may not currently address all the items on the checklist. Please modify your text to include the missing information. If you are certain that an item does not apply, please write "n/a" and provide a short explanation.

Upload your completed checklist as an extra file when you submit to a journal.

In your methods section, say that you used the STROBE cross sectionalreporting guidelines, and cite them as:

von Elm E, Altman DG, Egger M, Pocock SJ, Gotzsche PC, Vandenbroucke JP. The Strengthening the Reporting of Observational Studies in Epidemiology (STROBE) Statement: guidelines for reporting observational studies.

		Reporting Item	Page Number
Title and abstract			
Title	#1a	Indicate the study's design with a commonly used term in the title or the abstract	1
Abstract	#1b	Provide in the abstract an informative and balanced summary of what was done and what was found	1
Introduction			
Background / rationale	#2	Explain the scientific background and rationale for the investigation being reported	2-4
Objectives	#3	State specific objectives, including any prespecified hypotheses	4
Methods			
Study design	#4	Present key elements of study design early in the paper	4
Setting	#5	Describe the setting, locations, and relevant dates, including periods of recruitment, exposure, follow-up, and data collection	4

For peer review only

Eligibility criteria	#6a	Give the eligibility criteria, and the sources and methods of selection of participants.	4
	#7	Clearly define all outcomes, exposures, predictors, potential confounders, and effect modifiers. Give diagnostic criteria, if applicable	4-5
Data sources / measurement	#8	For each variable of interest give sources of data and details of methods of assessment (measurement). Describe comparability of assessment methods if there is more than one group. Give information separately for for exposed and unexposed groups if applicable.	4-5
Bias	#9	Describe any efforts to address potential sources of bias	
Study size	#10	Explain how the study size was arrived at	
Quantitative variables	#11	Explain how quantitative variables were handled in the analyses. If applicable, describe which groupings were chosen, and why	5-6
Statistical methods	#12a	Describe all statistical methods, including those used to control for confounding	5-6
Statistical methods	#12b	Describe any methods used to examine subgroups and interactions	5-6
Statistical methods	#12c	Explain how missing data were addressed	5-6
Statistical methods	#12d	If applicable, describe analytical methods taking account of sampling strategy	
Statistical methods	#12e	Describe any sensitivity analyses	5-6
Results			
Participants	#13a	Report numbers of individuals at each stage of study—eg numbers potentially eligible, examined for eligibility, confirmed eligible, included in the study, completing follow-up, and analysed. Give information separately for for exposed and unexposed groups if applicable.	6
Participants	#13b	Give reasons for non-participation at each stage	N/A
Participants	#13c	Consider use of a flow diagram	N/A
Descriptive data	#14a	Give characteristics of study participants (eg demographic, clinical, social) and information on exposures and potential confounders. Give information separately for exposed and unexposed groups if applicable.	6
Descriptive data	#14b	Indicate number of participants with missing data for each variable of interest	6

For peer review only

Outcome data	#15	Report numbers of outcome events or summary measures. Give information separately for exposed and unexposed groups if applicable.	N/A
Main results	#16a	Give unadjusted estimates and, if applicable, confounder-adjusted estimates and their precision (eg, 95% confidence interval). Make clear which confounders were adjusted for and why they were included	7-10
Main results	#16b	Report category boundaries when continuous variables were categorized	7-10
Main results	#16c	If relevant, consider translating estimates of relative risk into absolute risk for a meaningful time period	
Other analyses	#17	Report other analyses done—e.g., analyses of subgroups and interactions, and sensitivity analyses	
Discussion			
Key results	#18	Summarise key results with reference to study objectives	10
Limitations	#19	Discuss limitations of the study, taking into account sources of potential bias or imprecision. Discuss both direction and magnitude of any potential bias.	13
Interpretation	#20	Give a cautious overall interpretation considering objectives, limitations, multiplicity of analyses, results from similar studies, and other relevant evidence.	10-13
Generalisability	#21	Discuss the generalisability (external validity) of the study results	13
Other Information			
Funding	#22	Give the source of funding and the role of the funders for the present study and, if applicable, for the original study on which the present article is based	14

None The STROBE checklist is distributed under the terms of the Creative Commons Attribution License CC-BY. This checklist can be completed online using https://www.goodreports.org/, a tool made by the EQUATOR Network in collaboration with Penelope.ai

For peer review only

BMJ Open

The relationship between working conditions and psychological distress experienced by junior doctors in the UK during the COVID-19 pandemic: A cross-sectional survey study

Journal:	*BMJ Open*
Manuscript ID	bmjopen-2022-061331.R1
Article Type:	Original research
Date Submitted by the Author:	16-Jun-2022
Complete List of Authors:	Dunning, Alice; Bradford Teaching Hospitals NHS Foundation Trust, Research<br>Teoh, Kevin; Birkbeck University of London, Department of Organizational Psychology<br>Martin, James; University of Birmingham, Institute of Applied Health Research<br>Spiers, Johanna; University of Birmingham, College of Medical and Dental Sciences<br>Buszewicz, Marta; University College London, Research Department of Primary Care and Population Health<br>Chew-Graham, Carolyn; Keele University<br>Taylor, Anna; University of Leeds,<br>Gopfert, Anya; Oxford University Hospitals NHS Trust<br>Van Hove, Maria; London School of Hygiene & Tropical Medicine<br>Appleby, Louis; The University of Manchester Faculty of Medical and Human Sciences, Psychiatry & Behavioral Sciences<br>Riley, Ruth; University of Surrey, School of Health Sciences
**Primary Subject Heading**:	Mental health
Secondary Subject Heading:	Health services research
Keywords:	COVID-19, EDUCATION & TRAINING (see Medical Education & Training), Health & safety < HEALTH SERVICES ADMINISTRATION & MANAGEMENT, Human resource management < HEALTH SERVICES ADMINISTRATION & MANAGEMENT, Organisational development < HEALTH SERVICES ADMINISTRATION & MANAGEMENT, Quality in health care < HEALTH SERVICES ADMINISTRATION & MANAGEMENT

SCHOLARONE™
Manuscripts

I, the Submitting Author has the right to grant and does grant on behalf of all authors of the Work (as defined in the below author licence), an exclusive licence and/or a non-exclusive licence for contributions from authors who are: i) UK Crown employees; ii) where BMJ has agreed a CC-BY licence shall apply, and/or iii) in accordance with the terms applicable for US Federal Government officers or employees acting as part of their official duties; on a worldwide, perpetual, irrevocable, royalty-free basis to BMJ Publishing Group Ltd ("BMJ") its licensees and where the relevant Journal is co-owned by BMJ to the co-owners of the Journal, to publish the Work in this journal and any other BMJ products and to exploit all rights, as set out in our licence.

The Submitting Author accepts and understands that any supply made under these terms is made by BMJ to the Submitting Author unless you are acting as an employee on behalf of your employer or a postgraduate student of an affiliated institution which is paying any applicable article publishing charge ("APC") for Open Access articles. Where the Submitting Author wishes to make the Work available on an Open Access basis (and intends to pay the relevant APC), the terms of reuse of such Open Access shall be governed by a Creative Commons licence – details of these licences and which Creative Commons licence will apply to this Work are set out in our licence referred to above.

Other than as permitted in any relevant BMJ Author's Self Archiving Policies, I confirm this Work has not been accepted for publication elsewhere, is not being considered for publication elsewhere and does not duplicate material already published. I confirm all authors consent to publication of this Work and authorise the granting of this licence.

For peer review only

The relationship between working conditions and psychological distress experienced by junior doctors in the UK during the COVID-19 pandemic: A cross-sectional survey study

Authors: Alice Dunning, Kevin Rui-Han Teoh, James Martin, Johanna Spiers, Marta Buszewicz, Carolyn Chew-Graham, Anna Kathryn Taylor, Anya Gopfert, Maria Van Hove, Louis Appleby, Ruth Riley

Alice Dunning (Corresponding author)

Yorkshire Quality and Safety Research Group

Bradford Institute for Health research

Bradford Teaching Hospitals NHS Foundation Trust

Bradford

United Kingdom

Alice.dunning@bthft.nhs.uk

Kevin Rui-Han Teoh

Department of Organizational Psychology

Birkbeck, University of London

London

United Kingdom

James Martin

Institute of Applied Health Research

University of Birmingham

Birmingham

United Kingdom

Johanna Spiers

Institute of Applied Health Research

University of Birmingham

For peer review only

Birmingham

United Kingdom

Marta Buszewicz

Research Department of Primary Care and Population Health

University College London Medical School

London

United Kingdom

Carolyn A. Chew-Graham

School of Medicine

Keele University

Keele

United Kingdom

Anna K. Taylor

Leeds Institute of Health Sciences

Faculty of Medicine and Health

University of Leeds

Leeds

United Kingdom

Anya Gopfert

Torbay Council,

Torquay,

TQ1 3DR

Maria van Hove

The University of Exeter,

Exeter,

EX4 4PY

For peer review only

Louis Appleby

Division of Psychology and Mental Health

School of Medicine

University of Manchester

Manchester

United Kingdom

Ruth Riley

School of Health Sciences

University of Surrey

Faculty of Health and Medical Sciences

Guildford

Surrey

United Kingdom

For peer review only

Abstract

Objectives: This paper explored the self-reported prevalence of depression, anxiety and stress among junior doctors during the COVID-19 pandemic. It also reports the association between working conditions and psychological distress experienced by junior doctors.

Design: A cross-sectional online survey study was conducted, utilising the DASS-21 and HSE scale to measure psychological wellbeing and working cultures of junior doctors.

Setting: The National Health Service in the UK.

Participants: A sample of 456 UK junior doctors was recruited online during the COVID-19 pandemic from March 2020-January 2021.

Results: Junior doctors reported poor mental health, with over 40% scoring extremely severely depressed (45.2%), anxious (63.2%) and stressed (40.2%). Both gender and ethnicity were found to have a significant influence on levels of anxiety. Hierarchical multiple linear regression analysis

outlined the specific working conditions which significantly predicted depression (increased demands [β =.101], relationships [β = .27], unsupportive manager [β = -.111], anxiety (relationships [β = .31], change [β = .18], demands [β =.179] and stress (relationships [β = .18], demands [β = .28], role [β = .11]).

Conclusions: The findings illustrate the importance of working conditions for junior doctors' mental health, as they were significant predictors for depression, anxiety and stress. Therefore, if the mental health of junior doctors is to be improved, it is important that changes or interventions specifically target the working environment rather than factors within the individual clinician.

Article summary: Strengths and limitations

- The survey was delivered between March 2020 and January 2021, and therefore data was included both peak and non-peak in infection rates of the pandemic meaning the results are significant to the study aims.
- The study design links participants' psychological distress against an established and validated measure of working cultures.
- The sample of participants showed diverse representation across ethnicity, specialties and years of experience.
- However, the sample was not proportionately representative, as the majority of participants were white and female.

Background

The demands of workload and job expectations on healthcare workers have been widely acknowledged,[1]. These demands have been further exacerbated by the COVID-19 pandemic, which created unprecedented working conditions for these workers,[2]. Prior to the pandemic the 2019 National Health Service (NHS) staff survey found that 40% staff reported feeling unwell due to work-related stress in 5 years,[3]. The 2019 NHS Long Term Plan,[4] outlined the impact of increased

For peer review only

demands on healthcare staff wellbeing. Ten years previously, the NHS Health and Well-Being report argued that staff wellbeing should be at the centre of all organisations in the NHS,[5]. However, despite acknowledgment of the demanding work environments and poor wellbeing among healthcare workers in the NHS, relatively little has been done to better support staff,[6].

Doctors report more anxiety and depression than the general population,[7]. A particularly vulnerable group of doctors to experiencing distress are junior doctors. There are currently 65 542 junior doctors working in the NHS, [8], this includes qualified doctors in training or doctors who are not consultants; this title also includes foundation year doctors,[9]. The General Medical Council (GMC) has highlighted the low morale, distress and alienation experienced by junior doctors,[10]. Junior doctors and medical students reported the highest rates of being diagnosed with a mental illness, including anxiety and depression during the previous 12 months compared to others doctors; of these, 91% of junior doctors were at high or very high risk of burnout,[11]. Furthermore, Irish junior doctors reported significantly higher levels of psychological distress in comparison to consultants,[12]. Junior doctors' have also reported feeling dehumanised by their employers,[13]. Evidence suggests that junior doctors are more vulnerable to feeling distressed which are attributed to feelings of being unsupported, devalued, without autonomy,[14] A barrier for junior doctors seeking help for mental health concerns is the fear of failing if off sick,[15].

Research has identified gender differences in the mental health of UK doctors, in 2019 the gender spilt of all UK doctors was 45% female, 55% male, [16]. One survey found that females showed a higher risk of burnout and were 50% more likely to report a mental illness diagnosis compared to their male counterparts,[11]. Although there are mixed findings about suicide rates internationally, there are concerns over suicidality among doctors, particularly amongst female doctors,[17].

Frontline healthcare professionals have reported increased levels of psychological distress whilst working during the COVID-19 pandemic, [18- 23]. Furthermore, research has demonstrated the increased risk of burnout for NHS doctors at this time, with nearly half describing deterioration

For peer review only

of their mental health,[24]. High levels of psychological distress have been reported among anaesthesiology, emergency medicine and intensive care doctors in both Ireland and the UK,[25]. The increased work demands, redeployment, inadequate access to basic facilities or safe spaces to rest, loss of autonomy and anxiety around the plans for recovery were identified as contributing factors to the deterioration of their mental health,[24, 26]. The pandemic has also had a significant impact on training and further exacerbated the levels of poor mental health among junior doctors, with 70% experiencing anxiety, depression and burnout,[27].

Working conditions as a predictor of junior doctors' mental health

In light of the prevalence of poor mental health among junior doctors, it is important to understand the relevant antecedent factors. While workplace health interventions typically focus on addressing individual-level factors in the behaviour or approach of junior doctors (e.g., coping behaviours and resilience), there is increasing recognition that how workplaces are organised, designed and managed are more important contributing factors, not only to doctors' mental health but also towards patient care,[28, 29]. Two work-related factors, including high demands and a poor environment, were identified as being associated with higher levels of burnout among junior doctors within a systematic review of 48 studies,[30]. An Australian survey found that increasing working hours led to an increase in mental health problems among junior doctors,[7]. Qualitative research in Australia identified support from colleagues as improving junior doctors' levels of burnout and wellbeing,[31]. Similarly, interviews with junior doctors in the UK identified poor working conditions, toxic work cultures, lack of support and mental health stigma as being contributing factors to poor levels of wellbeing among junior doctors,[32].

To better, and more holistically, support the mental health of junior doctors it is important to understand the specific working conditions within the UK health system related to their mental health. Although other studies have correlated other measures of work environment with the mental health of NHS doctors (e.g.,[28]) or nurses,[33, 34], this has not been done specifically for junior doctors.

For peer review only

Aims

To explore the prevalence of depression, anxiety and stress experienced by junior doctors in the UK during the COVID-19 pandemic.

To identify working conditions associated with psychological distress.

Method

Study design and sample

A cross-sectional online survey, conducted as part of a wider mixed methods study,[31, 35], was utilised to measure the working conditions and mental health of a convenience sample of UK junior doctors. Participants were recruited online from March 2020 until January 2021 through advertisements on social media posts, junior doctor forums and emails circulated via specialist schools. The survey was hosted on the online platform Qualtrics, which allowed participants to access the questionnaire at a time which suited them. Participants were entered into a prize draw for shopping vouchers (three prizes of £50 were available) to thank them for their time.

Measures

Demographic information

Age, gender, ethnicity, sexuality, years as a doctor and speciality were recorded at the beginning of the online survey.

Mental Health

Levels of depression, anxiety and stress were all measured using the 21-item Depression, Anxiety and Stress Scale (DASS-21),[36]. Participants rated the extent to which they had experienced each item within the past week on a four-point Likert scale. Following the scale guidelines, participant scores for depression, anxiety and stress were multiplied by two, and categorised into normal, mild, moderate, severe and extremely severe, following the recommended cut-off scores.

Working conditions

The UK Health and Safety Executive's (HSE) management standards framework was utilised. This identifies seven key aspects of the working environment using a 35-item scale: role clarity; peer

For peer review only

support; strained relationships; managerial support; work demands; control over working environment; and change. These are based on relevant occupational health psychology theories (e.g., the job demands resources model),[37]. While the framework has been widely applied across different sectors (e.g.,[38-40]), we are not aware of its application in the NHS. Participants were asked to rate how often they experienced each item within the last six months on a five-point Likert scale.

Ethical considerations

Participants were given an information sheet and asked to provide online consent prior to responding to the survey. All identifiable information was stored securely and separately to non-identifiable survey data. The research was approved by the University of Birmingham ethics committee and Health Research Authority (reference number: 19/HRA/6579)

Patient and Public Involvement

No patient involvement.

Data Analysis

After data was extracted from Qualtrics, we carried out data management and analysis using SPSS version 26. Measures were scored according to their instructions and, where appropriate, items reverse scored so that a higher score represents a stronger measure of the construct. Participant demographics were summarised using numbers and percentages, means and standard deviations or median and inter-quartile ranges, as appropriate. A Shapiro-Wilk test was used to assess normality. The normality of the variables was not established as all the measures were skewed and had a kurtosis score greater than the absolute value of 2.0. Little's chi-square statistic for testing whether values are missing completely at random (MCAR),[41] was not significant (X^2 (1746, 456) = 1145.09, p=1.00). This demonstrates that there was no pattern to the missing values within the data set. To test for association between participants' characteristics and the depression, anxiety, and stress scores, we used Kruskall-Wallis non-parametric tests with Bonferonni

For peer review only

adjustments to test against the raw scores, and chi-squared tests to test against the classified scores. We then carried out Spearman's Rho correlations between the study variables as assumptions of normality were not met.

To quantify the effect of working conditions on each of the outcomes (depression, anxiety, and stress), we fitted two linear regression models to each outcome. Our first model included only the participants' demographics (age, gender, years as a doctor). The second model included participants' demographics and the seven working conditions (demands, control, manager support, peer support, role, change, strained relationships).

Results

Participant characteristics and levels of depression, anxiety and stress

A total of 456 responses were received with the mean age of 30.70 (*SD* = 4.87). The mean number of years working as a doctor was 4.94 (*SD* = 3.56). Most participants identified as female (67.1%) and came from a white background (71.1%), 80% of the sample identified as heterosexual. A full demographic breakdown is presented in Table 1.

For peer review only

Table 1. Frequency and percentages of participant demographics mapped against degree of depression, anxiety, and stress.

	N	Depression					Anxiety					Stress					
		Normal / Mild	Moderate	Severe	Extremely Severe	*p* value	Normal/ Mild	Moderate	Severe	Extremely Severe	*p* value	Normal	Mild	Moderate	Severe	Extremely severe	*p* value
Total	456 (100%)	0 (0%)	135 (31.4%)	100 (23.3%)	194 (45.2%)	.01*	0 (0%)	50 (11.7%)	108 (25.2%)	271 (63.2%)	.01*	8 (1.9%)	39 (9.1%)	74 (17.3%)	135 (31.5%)	172 (40.2%)	.01*
Gender						.78					.22						.67
Male	123 (27%)	-	33 (28.7%)	30 (26.1%)	52 (45.2%)		-	16 (13.9%)	35 (30.4%)	64 (55.7%)		3 (2.6%)	11 (9.6%)	25 (21.7%)	33 (28.7%)	43 (37.4%)	
Female	306 (67.1%)	-	96 (33.1%)	65 (22.4%)	129 (44.5%)		-	31 (10.7%)	72 (24.8%)	187 (64.5%)		4 (1.4%)	28 (9.7%)	45 (15.6%)	97 (33.6%)	115 (39.8%)	
Prefer to self-describe	4 (0.9%)	-	1 (33.9%)	1 (33.9%)	1 (33.9%)		-	-	-	3 (100%)		-	-	1 (33.9%)	1 (33.9%)	1 (33.9%)	
Prefer not to say	23 (5%)	-	1 (14.3%)	1 (14.3%)	5 (71.4%)		-	2 (28.6%)	-	5 (71.4%)		-	-	2 (28.6%)	-	5 (71.4%)	
Ethnicity						.28					.07						.93
White	324 (71.1%)	-	100 (32.8%)	72 (23.6%)	133 (43.6%)		-	39 (12.8%)	83 (27.2%)	183 (60%)		7 (2.3%)	27 (8.9%)	53 (17.4%)	99 (32.6%)	118 (38.8%)	
Mixed	26 (6/4%)	-	10 (38.5%)	8 (30.8%)	8 (30.8%)		-	2 (7.7%)	9 (34.6%)	15 (57.7%)		-	3 (11.5%)	3 (11.5%)	10 (38.5%)	10 (38.5%)	
Asian	72 (15.8%)	-	18 (26.1%)	16 (23.2%)	35 (50.7%)		-	4 (5.8%)	11 (15.9%)	54 (78.3%)		1 (12.5%)	7 (10.1%)	10 (14.5%)	21 (30.4%)	30 (43.5%)	
Black	9 (2%)	-	3 (37.5%)	2 (25%)	3 (37.5%)		-	-	2 (25%)	6 (75%)		-	1 (12.5%)	2 (25%)	2 (25%)	3 (37.5%)	
Other	25 (5.5%)	-	4 (19%)	2 (9.5%)	15 (71.4%)		-	5 (23.8%)	3 (14.3%)	13 (61.9%)		-	1 (4.8%)	6 (28.6%)	3 (14.3%)	11 (52.4%)	
Sexuality						.42					.49						.17

For peer review only

Heterosexual	365 (80%)	-	113 (32.8%)	79 (23%)	152 (44.2%)	-	43 (12.5%)	89 (25.9%)	212 (61.6%)	8 (2.3%)	33 (84.6%)	57 (31.2%)	107 (31.2%)	138 (40.2%)
LGBTQ+	69 (15.2%)	-	18 (27.7%)	18 (27.7%)	29 (44.6%)	-	4 (6.2%)	17 (26.2%)	44 (67.7%)	-	5 (7.7%)	10 (15.4%)	26 (40%)	24 (36.9%)
Prefer not to say	21 (4.6%)	-	3 (20%)	2 (13.3%)	10 (66.7%)	-	2 (13.3%)	2 (13.3%)	11 (73.3%)	-	1 (6.7%)	6 (40%)	1 (6.7%)	7 (46.7%)

For peer review only

Table 1 provides an overview of the proportion of the sample that were categorised on each degree of the mental health measures. Across all three measures, at least 70% of the sample scored 'severe' or 'extremely severe'. For depression, 45.2% of participants were classed as having extremely severe levels of depression, with an additional 23.3% classed as severe ($p<.001$). There were more participants classed as having extremely severe levels of anxiety (63.2%) compared to those with severe (25.2%) or moderate (11.7%) levels ($p<0.001$). No participants had normal or mild levels of depression or anxiety. More participants also reported extremely severe (40.2%) or severe (31.5%) levels of stress than less severe levels ($p<.001$). Chi-square analysis between participant characteristics (i.e., gender, ethnicity, sexuality) and classification of depression, anxiety and stress showed no significant associations (Table 1).

Table 2: Mean comparisons between participants' characteristics for levels of depression, anxiety and stress.

	Depression			Anxiety			Stress		
Characteristic	Mean	*S.D.*	*p* value	Mean	*S.D.*	*p* value	Mean	*S.D.*	*p* value
Gender	28.21	11.30	.44	24.94	10.32	.03	30.97	9.60	.29
Male	28.91	11.47		23.71	10.58		30.37	10.25	
Female	27.92	11.23		25.45	10.18		31.21	9.32	
Ethnicity	28.50	11.47	.01	25.15	10.41	.01	31.14	9.63	.77
White	27.74	27.80		24.16	9.77		30.81	9.45	
Asian	31.51	12.84		27.88	11.42		31.82	10.08	
Black	26.75	10.79		32.50	13.17		31.00	11.76	
Mixed	24.80	9.48		25.04	20.23		30.72	8.18	
Other	34.76	12.70		28.00	12.79		34.19	11.52	
Sexuality	28.51	11.52	.10	25.12	10.43	.26	31.10	9.64	.66
Heterosexual	28.17	11.37		24.76	10.24		30.90	9.73	
LGBTQ+	28.83	11.88		26.21	10.93		32.12	9.15	

For peer review only

Prefer not to say	34.93	12.23	28.67	12.32	31.20	9.97

Note. *p* values are for differences between means within each characteristic

We found no evidence of differences on levels of depression (*p*=.439) and stress (*p*=.290) between male and female participants (Table 3). However, significant differences were reported for anxiety (*p*=.032), were participants who identified as female (*M* = 25.45) reported a higher mean score for anxiety than male (*M* = 23.71). No differences were observed based on their sexual orientation for levels of depression (*p*=.098), anxiety (*p*=.260), or stress (*p*=.662).

In terms of ethnicities, a Kruskall-Wallis (Table 2) found a significant difference in levels of depression (*p*=.016). Post-hoc comparison using Bonferroni test showed participants from mixed (*M* = 24.80) background reporting lower levels of depression than those from an Asian (*M* = 31.51) or White (*M* = 27.74) background. Participants from a White (*M* = 27.74) background also reported lower levels of a depression than those from an Other (*M* = 34.76) and from an Asian (*M* = 31.55) background. Differences were also observed across ethnicities for anxiety (*p*=.020), with Bonferroni comparisons showing that participants from an Asian background (*M* = 27.88) reported higher levels of anxiety than those from a white background (*M* = 24.16). No differences were observed for stress (*p* = .770) between participants from different ethnic groups.

Descriptive statistics and relationships for perceived working conditions, depression, anxiety, and stress

Table 3 presents the descriptive statistics and internal reliabilities for all variables in this study, and that all composite measures had adequate internal consistency (α >.70). Bivariate two-tailed non-parametric correlations show the presence of all anticipated relationships in the appropriate directions. More specifically, reporting more challenging working conditions (i.e., high demands, strained relationships) were was associated with lower levels of positive working conditions (i.e., control, manager support, peer support, role, change), as well as higher levels of

For peer review only

depression, anxiety and stress. Higher levels of the four positive working conditions were correlated with lower levels of junior doctors' levels of depression, anxiety and stress.

For peer review only

Table 3: Descriptive statistics, correlations, and reliability coefficients among study variables

Variable	Internal consistency (Cronbach α)	1	2	3	4	5	6	7	8	9	10	11	12
1. Years as doctor	-	4.94 (3.56)	.70**	-.09*	.15**	.10*	.04	.04	.20**	.09	-.02	-.17**	-.03
2. Age	-		30.70 (4.87)	-.04	.10*	.04	-.06	.07	.06	-.01	.03	-.16**	-.04
3. Demands	.86			3.19 (0.68)	-.60**	-.52**	-.45**	.53**	-.42**	-.43**	.40**	.34**	.45**
4. Control	.84				2.65 (0.71)	.66**	.52**	-.42**	.43**	.58**	-.37**	-.29**	-.34**
5. Manager support	.90					2.98 (0.95)	.71**	-.54**	.51**	.66**	-.46**	-.30**	-.39**
6. Peer support	.84						3.61 (0.73)	-.56**	.48**	.46**	-.45**	-.32**	-.38**
7. Strained Relationships	.84							2.34 (0.)	-.45**	-.41**	.49**	.36**	.42**
8. Role	.74								3.80 (0.58)	.46**	-.36**	-.25**	-.35**
9. Change	.80									2.42 (0.81)	-.37**	-.20**	-.33**
10. Depression	.93										28.50 (11.47)	.56**	.72**
11. Anxiety	.79											25.15 (10.41)	.70**
12. Stress	.88												31.14 (9.63)

Note. Mean (*SD*) are reported diagonally; ***p*<.01 level (2-tailed). **p*<.05 level (2-taile)

For peer review only

Direct effects between perceived working conditions and levels of depression, anxiety and stress

Table 4 presents report that junior doctors' strained relationships (β = .27) positively predicted levels of depression. Having good peer support was negatively associated with levels of depression (β = -.15). There was no evidence of an association between participants' characteristics (age, gender, and years as a doctor) and depression.

Table 4: Multiple linear regression analyses with depression, anxiety and stress as dependant variables

	Step 1		Step 2	
Predictors	β	B [95% CI]	β	B [95% CI]
Depression				
Age	.048	0.110 (-0.216, 0.435)	.007	0.017 (-0.259, 0.292)
Gender (Female)	-.027	0.675 (-3.156, 1.807)	-.012	-0.305 (-2.400, 1.791)
Years as doctor	-.036	-0.110 (-.0.543, 0.324)	.004	0.014 (-0.359, 0.386)
Demands			.101	1.683 (-0.187, 3.552)
Control			-.017	-.273 (-2.245, 1.699)
Manager support			-.111	-1.323 (-3.066, 0.420)
Peer			-.147	-2.266 (-4.251, -0.281)
Strained relationships			.273	3.954 (2.314, 5.593)
Role			-.088	-1.699 (-3.775, 0.377)
Change			.039	.550 (-1.107, 2.208)
R^2		0.002		0.324
ΔR^2		0.002		0.322
p value		0.845		0.001
Anxiety				
Gender (Female)	0.079	1.784 (-0.443, 4.000)	.108	2.457 (0.458, 4.457)
Years as doctor	-0.095	-0.266 (0.653, 0.122)	-.083	-.233 (-0.589, 0.122)
Age	-0.088	-0.184 (-0.475, 0.106)	-.120	-.251 (-0.514, 0.012)
Demands			.179	2.716 (0.932, 4.500)
Control			.028	.413 (-1.469, 2.295)
Manager support			.009	.100 (-1.563, 1.763)
Peer			-.105	-1.476 (-3.369, 0.418)

For peer review only

Strained relationships			.314	4.135 (2.571, 5.699)
Role			-.051	-.886 (-2.867, 1.095)
Change			.104	1.328 (-0.3254, 2.909)
R^2		0.037		0.256
ΔR^2		0.037		0.219
p value		0.002		0.001
Stress				
Gender (Female)	.049	1.029 (-1.081, 3.140)	.061	1.298 (-0.511, 3.108)
Years as doctor	-.034	-0.09 (-0.459, 0.279)	.022	.057 (-0.266, 0.379)
Age	.019	0.038 (-0.239, 0.314)	-.027	-.054 (-0.291, 0.184)
Demands			.294	4.164 (2.549, 5.479)
Control			.065	.879 (-0.824, 2.582)
Manager support			-.034	-.346 (-1.851, 1.160)
Peer			-.103	-1.352 (-3.067, 0.363)
Strained relationships			.181	2.225 (0.807, 3.644)
Role			-.119	-1.943 (-3.735, -0.151)
Change			-.007	-.089 (-1.521, 1.343)
R^2		0.003		0.304
ΔR^2		0.003		0.301
p value		0.764		0.001

Note. * *p* < .05, ** *p* < .01; CI: 95% unstandardised confidence intervals.

For anxiety, junior doctors who identified as female reported higher levels of anxiety (β = .11) than those who identified as male. Table 4 shows that strained relationships were the strongest predictor (β = .31) of anxiety, followed by how well change is managed (β = .18.

Focusing on stress, we see that demographic factors made no contribution to the explained variance in levels of stress. Strained relationships (β = .18) and demands (β = .28) positively predicted stress. In addition, role clarity was associated with lower levels of stress (β = .11).

Discussion

The findings show that junior doctor survey respondents reported high levels of depression, anxiety, and stress during the pandemic, with at least 70% reporting 'severe' or 'extremely severe'

For peer review only

across each measure. This study expands what has already been described in the literature by showing that specific working conditions are associated with junior doctors' levels of psychological distress, namely depression (strained relationships, managerial support), anxiety (strained relationships, organisational change, high demands) and stress (strained relationships, high demands and role).

The high levels of depression anxiety and stress which were reported by junior doctors in this study build upon previous literature which reports elevated psychological distress among junior doctors as compared to pre-pandemic levels,[12]. Crucially, these figures are higher than pre-pandemic comparisons of samples including doctors in the NHS,[42] and Ireland,[11] reflecting the increased strain which healthcare staff experienced whilst working on the frontline,[2, 24, 27]

Furthermore, female junior doctors in this study reported higher levels of anxiety compared with their male counterparts. As increased levels of anxiety are associated with suicidality,[43] this link may provide some insight into the elevated risk of suicide among female doctors,[12]. This difference in anxiety between females and males may be explained by work-related factors such as poorer work life balance,[13]. Further research should explore levels of suicidality among junior doctors as well as potential gender differences. There were also significant differences between levels of anxiety experienced by different ethnicities within this study, with junior doctors from an Asian background reporting higher levels than junior doctors from a White background. While no significant differences were observed for other ethnic groups, ethnic minorities in almost all instances reported poorer levels of mental health. It may be that the lack of statistical power led to the lack of more significant differences being found. This finding is supported by previous literature, which outlines the increased risk of mental health problems for ethnic minorities,[44]. This study is the first to demonstrate an elevated risk of poor mental health among junior doctors an Asian background. This difference between ethnicities may be explained by poorer working conditions which are experienced by ethnic minorities ,[45] or the increased likelihood of experiencing harassment in the workplace,[32, 46]. Future research should explore whether there are additional

For peer review only

factors associated with differences between gender, ethnicity, and anxiety; such data could support targeted changes or interventions to support any at risk group.

Working conditions as an antecedent to junior doctors' mental ill-health

The most consistent predictor of depression, anxiety and stress was strained relationships. Previous literature supports this finding, as bullying or witnessing harassment has been found to impact NHS healthcare staff wellbeing and job satisfaction,[47]. Furthermore, toxic working cultures, including bullying, discrimination and a blame culture, were found to be the most frequently discussed source of distress for junior doctors,[32]. A lack of supportive colleagues during incidents or through discontinuity in teams was also highlighted by Riley and colleagues,[35] as a contributing factor to psychological distress in junior doctors.

Additionally, an online survey found the most reported factor for boosting junior doctor morale in the UK was feeling part of a team,[48] demonstrating the impact positive relationships can have. Further, belonging, feeling valued and connected with colleagues and supervisors/consultants/mangers is protective and is associated with better morale and positive mental health among staff,[47]. In line with these findings, interventions which centre on creating supportive working relationships, such as peer mentoring programmes, have been found to have a positive effect on psychological wellbeing and job satisfaction for junior doctors,[48].

Belonging, feeling valued and connectedness are protective factors and can promote positive mental health and a positive professional identity; for instance, peer/colleague support in the workplace has been found to mitigate the negative psychological consequences of adverse events. In line with the evidence that supportive relationships with colleagues are important for mitigating psychological distress, supportive managers were found to be significantly associated with lowerlevels of depression in this study. The importance of supportive leadership for healthcare staff,[49] and doctors',[29] wellbeing has been previously identified. Supportive relationships and feeling valued by supervisors/consultants acts a buffer against the demands of the job,[35]. Taken

For peer review only

together, the findings that strained relationships and supportive leadership are associated with mental health demonstrate the need for a supportive environment, in which junior doctors' work within consistent teams under good leadership to be adopted.

Intense work demands was also associated with psychological distress, demonstrating its importance for junior doctor wellbeing. Previous literature has outlined the increasing strain on healthcare workers because of the demands of their role,[1] which were exacerbated through the pandemic,[2, 24]. Considering the demands healthcare staff face, the prevalence of poor mental health found in this study is unsurprising. This association between high/intense job demands and poor mental health is supported by previous research where job demands predicted doctors' reported wellbeing ,[50] were associated with junior doctors' levels of burnout,[30] and contributed to junior doctors' psychological distress,[32]. This research further highlights the need to address the demanding working conditions which are impacting on junior doctors' distress.

A key strength of this study is the diverse representation across ethnicities, different specialities and years of experience. However, most of the junior doctors in this study were white females. Considering the gender and ethnicity differences reported in the results, a more proportionally representative sample would have improved the generalisability of the results. Furthermore, the use of convenience sampling recruitment methods, the results may be biased to junior doctors who access online platforms. We did not consider positive manifestations of wellbeing (e.g., job satisfaction, work engagement) which have been linked to both doctors' working environment and to patient care, [51. 52]. This was beyond the scope of this funded study, but future researchers should explore a more rounded perspective of doctors' wellbeing. Finally, the cross-sectional study design means causality cannot be presumed, and the single time-point measure also does not capture the full effects of the pandemic, with other studies showing a general deterioration of healthcare workers mental health over the course of the pandemic, [26, 53, 54]. As we did not factor in date of completion, we were not able to determine the proportion of respondents who completed the survey during one of the peak periods. Nonetheless, the findings

For peer review only - http://bmjopen.bmj.com/site/about/guidelines.xhtml

from this study provide a valuable understanding of the relationship between working conditions and mental health for junior doctors.

Conclusion

In summary, the findings of this study reflect the poor levels of psychological wellbeing many junior doctors' experienced during the pandemic. Our results demonstrate the association between working conditions, specifically job demands, strained relationships, organisational change and role certainty and the mental health of junior doctors. This indicates the need for systemic changes or interventions that focus on improving the working conditions of junior doctors rather than focusing on 'resilience' in the individual.

For peer review only

References

1 Lacobucci, G. (2015). Staff burnout threatens plans for NHS, says think tank. *BMJ: British Medical Journal (Online)*, *350*.

2 Greenberg, N., & Tracy, D. (2020). What healthcare leaders need to do to protect the psychological well-being of frontline staff in the COVID-19 pandemic. *BMJ Leader*.

3 NHS Staff Survey (2017). National Briefing Published March 2018. NHS Survey Coordination Centre.

4 Department of Health (2019), The NHS Long Term Plan, Available at: https://www.longtermplan.nhs.uk/wp-content/uploads/2019/08/nhs-long-term-planversion-1.2.pdf [accessed August 2020]

5 Boorman S (2009), NHS Health and Well-being Review: Interim Report: Available at: https://webarchive.nationalarchives.gov.uk/20130103004910/http://www.dh.gov.uk/en /Publicationsandstatistics/Publications/PublicationsPolicyAndGuidance/DH_108799 [accessed August 2020]

6 Bajorek, Z., & Holmes, J. (2020). Health and Wellbeing Interventions in Healthcare.

7 Petrie, K., Crawford, J., LaMontagne, A. D., Milner, A., Dean, J., Veness, B. G., ... & Harvey, S. B. (2020). Working hours, common mental disorder and suicidal ideation among junior doctors in Australia: a cross-sectional survey. *BMJ open*, *10*(1), e033525.

8 NHS Digital. NHS Workforce Statistics - January 2020, in NHS Hospital & Community Health Service (HCHS) monthly workforce statistics. 2020, Health and Social Care Information Centre, 2020. Available: https://digital.nhs.uk/data-and-information/publications/ statistical/nhs-workforce-statistics/january-2020

9 BMA, 2020. Available: https://www.bma.org.uk/advice-and-support/ international-doctors/life-and-work-in-the-uk/toolkit-for-doctorsnew-to-the-uk/doctors-titles-explained

10 GMC. Working with doctors for patients- 2016 Available at: https://www.gmc-uk.org/-/media/documents/GMC_Annual_Report_2016_web.pdf_71778870.pdf

For peer review only

11 Bhugra, D., Sauerteig, S. O., Bland, D., Lloyd-Kendall, A., Wijesuriya, J., Singh, G., ... & Ventriglio, A. (2019). A descriptive study of mental health and wellbeing of doctors and medical students in the UK. *International Review of Psychiatry*, *31*(7-8), 563-568.

12 Hayes, B., et al., *What's up doc? A national cross-sectional study of psychological wellbeing of hospital doctors in Ireland.* BMJ Open, 2017. **7**(10).

13 Rich, A., Viney, R., Needleman, S., Griffin, A., & Woolf, K. (2016). 'You can't be a person and a doctor': the work–life balance of doctors in training—a qualitative study. *BMJ open*, *6*(12), e013897.

14 NHS., *Junior doctor morale. Understanding best practice working environments*. 2017, Health Education England.

15 Fox, F. E., Doran, N. J., Rodham, K. J., Taylor, G. J., Harris, M. F., & O'Connor, M. (2011). Junior doctors' experiences of personal illness: a qualitative study. *Medical education*, *45*(12), 1251-1261.

16 NHS Digital. NHS Workforce Statistics – October 2019, NHS Hospital and Community Health Services: HCHS Doctors by specialty, gender and customer specified grade, in NHS Trusts and CCG, Health and Social Care Information Centre, 2019. Available: https://digital.nhs.uk/data-and-information/find-data-and-publications/supplementary-information/2019-supplementary-information-files/hshs-doctors-by-specialty-and-gender-ah3018

17 Office for National Statistics, 2017. Suicides in Great Britain: 2016 Registrations. https://www.ons.gov.uk/releases/suicidesingreatbritain2016 [accessed August 2020]

18 De Kock, J. H., Latham, H. A., Leslie, S. J., Grindle, M., Munoz, S. A., Ellis, L., ... & O'Malley, C. M. (2021). A rapid review of the impact of COVID-19 on the mental health of healthcare workers: implications for supporting psychological well-being. BMC public health, 21(1), 1-18.

19 Domínguez-Salas, S., Gómez-Salgado, J., Guillén-Gestoso, C., Romero-Martín, M., Ortega-Moreno, M., & Ruiz-Frutos, C. (2021). Health care workers' protection and psychological safety during the COVID-19 pandemic in Spain. *Journal of nursing management*, *29*(7), 1924–1933.

20 García-Iglesias JJ, Gómez-Salgado J, Martín-Pereira J, Fagundo-Rivera J, Ayuso-Murillo D, Martínez-Riera JR, Ruiz-Frutos C. Impacto del SARS-CoV-2 (Covid-19) en la salud mental de los

For peer review only

profesionales sanitarios: una revisión sistemática [Impact of SARS-CoV-2 (Covid-19) on the mental health of healthcare professionals: a systematic review.]. Rev Esp Salud Publica. 2020 Jul 23;94:e202007088. Spanish. PMID: 32699204.

21 Greenberg, N., Weston, D., Hall, C., Caulfield, T., Williamson, V., & Fong, K. (2021). Mental health of staff working in intensive care during COVID-19. *Occupational Medicine*, *71*(2), 62-67.

22 Gómez-Salgado J, Domínguez-Salas S, Romero-Martín M, Romero A, Coronado-Vázquez V, Ruiz-Frutos C. Work engagement and psychological distress of health professionals during the COVID-19 pandemic*. J Nurs Manag*. 2021 Jul;29(5):1016-1025.

23 Shacham, M., Hamama-Raz, Y., Kolerman, R., Mijiritsky, O., Ben-Ezra, M., & Mijiritsky, E. (2020). COVID-19 factors and psychological factors associated with elevated psychological distress among dentists and dental hygienists in Israel. *International journal of environmental research and public health*, 17(8), 2900

24 Cubitt, L. J., Im, Y. R., Scott, C. J., Jeynes, L. C., & Molyneux, P. D. (2021). Beyond PPE: a mixed qualitative–quantitative study capturing the wider issues affecting doctors' well-being during the COVID-19 pandemic. *BMJ open*, *11*(3), e050223.

25 Roberts, T., Daniels, J., Hulme, W., Hirst, R., Horner, D., Lyttle, M. D., ... & Carlton, E. (2021). Psychological distress during the acceleration phase of the COVID-19 pandemic: a survey of doctors practising in emergency medicine, anaesthesia and intensive care medicine in the UK and Ireland. *Emergency Medicine Journal*, *38*(6), 450-459

26 Spiers, J., Buszewicz, M., Chew-Graham, C., Dunning, A., Taylor, A. K., Gopfert, A., ... & Riley, R. (2021). What challenges did junior doctors face while working during the COVID-19 pandemic? A qualitative study. *BMJ open*, *11*(12), e056122.

27 Dekker, A., Lavender, D., Clarke, D., & Tambe, A. (2020). How has the COVID-19 pandemic affected junior doctor training? A survey analysis. *The Transient Journal of Trauma, Orthopaedics and the Coronavirus*.

For peer review only

28 De Lange, A. H., Løvseth, L. T., Teoh, K. R. H., & Christensen, M. (2020). Healthy healthcare: empirical occupational health research and evidence-based practice. *Frontiers in Psychology*, *11*, 2236.

29 Teoh, K. R. H., Hassard, J., & Cox, T. (2021). Doctors' working conditions, wellbeing and hospital quality of care: A multilevel analysis. *Safety Science*, *135*, 105115.

30 Zhou, A. Y., Panagioti, M., Esmail, A., Agius, R., Van Tongeren, M., & Bower, P. (2020). Factors associated with burnout and stress in trainee physicians: a systematic review and meta-analysis. *JAMA network open*, *3*(8), e2013761-e2013761.

31 Hoffman, R., & Bonney, A. (2018). Junior doctors, burnout and wellbeing: Understanding the experience of burnout in general practice registrars and hospital equivalents. *Australian journal of general practice*, *47*(8), 571-575.

32 Riley, R., Buszewicz, M., Kokab, F., Teoh, K., Gopfert, A., Taylor, A. K., ... & Chew-Graham, C. (2021). Sources of work-related psychological distress experienced by UK-wide foundation and junior doctors: a qualitative study. *BMJ open*, *11*(6), e043521.

33 Brunetto Y, Xerri M, Trinchero E, et al. Comparing the impact of management on public and private sector nurses in the UK, Italy, and Australia. Public Manag Rev. 2018;20(4):525-544.

34 Yoshida Y, Sandall J. Occupational burnout and work factors in community and hospital midwives: A survey analysis. Midwifery. 2013;29(8):921-926.

35 Riley, R., Kokab, F., Buszewicz, M., Gopfert, A., Van Hove, M., Taylor, A. K., ... & Chew-Graham, C. (2021). Protective factors and sources of support in the workplace as experienced by UK foundation and junior doctors: a qualitative study. *BMJ open*, *11*(6), e045588.

36 Lovibond, S. H., & Lovibond, P. F. (1996). *Manual for the depression anxiety stress scales*. Psychology Foundation of Australia.

37 Bakker, A. B., & Demerouti, E. (2017). Job demands–resources theory: taking stock and looking forward. *Journal of occupational health psychology*, *22*(3), 273.

For peer review only

38 Houdmont, J., Kerr, R., & Randall, R. (2012). Organisational psychosocial hazard exposures in UK policing: Management Standards Indicator Tool reference values. *Policing: An International Journal of Police Strategies & Management*.

39 Kinman, G., Clements, A. J., & Hart, J. (2017). Job demands, resources and mental health in UK prison officers. *Occupational Medicine*, *67*(6), 456-460.

40 Wattie, S. C., & Bridger, R. S. (2019). Work-related stress indicator surveys in UK Ministry of Defence. *BMJ Military Health*, *165*(2), 128-132.

41 Little, R. J. (1988). A test of missing completely at random for multivariate data with missing values. *Journal of the American statistical Association*, *83*(404), 1198-1202.

42 Cramer, R. J., Ireland, J. L., Hartley, V., Long, M. M., Ireland, C. A., & Wilkins, T. (2020). Coping, mental health, and subjective well-being among mental health staff working in secure forensic psychiatric settings: Results from a workplace health assessment. *Psychological services*, *17*(2), 160.

43 Thibodeau, M. A., Welch, P. G., Sareen, J., & Asmundson, G. J. (2013). Anxiety disorders are independently associated with suicide ideation and attempts: propensity score matching in two epidemiological samples. *Depression and anxiety*, *30*(10), 947-954.

44 Maura, J., & de Mamani, A. W. (2017). Mental health disparities, treatment engagement, and attrition among racial/ethnic minorities with severe mental illness: A review. *Journal of Clinical Psychology in Medical Settings*, *24*(3), 187-210.

45 NHS., *Workforce Race Equality Standard report*. 2021, NHS England

46 Rhead, R. D., Chui, Z., Bakolis, I., Gazard, B., Harwood, H., MacCrimmon, S., ... & Hatch, S. L. (2021). Impact of workplace discrimination and harassment among National Health Service staff working in London trusts: results from the TIDES study. *BJPsych open*, *7*(1).)

47 Carter M, Thompson N, Crampton P, et al. Workplace bullying in the UK NHS: a questionnaire and interview study on prevalence, impact and barriers to reporting. BMJ Open 2013;3:e002628

48 Singh, R., Kirtley, J., Minhas, J. S., Lakhani, D., & Carr, S. (2019). Exploring junior doctor morale in a UK hospital. *The journal of the Royal College of Physicians of Edinburgh*, *49*(4), 312-316.

For peer review only

49 Borrill C, West M, Shapiro D, et al. Team working and effectiveness in health care. British Journal of Healthcare Management 2000;6:364–71

50 Teoh, K. R. H., Hassard, J., & Cox, T. (2021). Doctors' working conditions, wellbeing and hospital quality of care: A multilevel analysis. *Safety Science*, *135*, 105115.

51 Montgomery, A. J. (2020). Well-Being, Patient Safety and Organizational Change: Quo Vadis?. In *Connecting Healthcare Worker Well-Being, Patient Safety and Organisational Change* (pp. 345-350). Springer, Cham.

52 Teoh, K. R.-H., Hassard, J., & Cox, T. (2020). Individual and organizational psychosocial predictors of hospital doctors' work-related well-being. *Health Care Management Review*, *45*(2), 162–172.

53 Gordon, L., Scanlan, G. M., Tooman, T. R., Walker, K. A., Cairns, P., Ferguson, J., ... & Wakeling, J. (2021). Heard, valued, supported? Doctors' wellbeing during transitions triggered by COVID-19. *Medical Education*.

54 López Steinmetz, L. C., Herrera, C. R., Fong, S. B., & Godoy, J. C. (2022). A Longitudinal Study on the Changes in Mental Health of Healthcare Workers during the COVID-19 Pandemic. *Psychiatry*, *85*(1), 56–71.

For peer review only

Author Contribution: AD KT JM JS MB CC-G AT AG MH LA and RR contributed to the study conception, design, data collection and approval of final article. AD KT JM and RR conducted the data analysis and drafting of the article. AD submitted the final version of this article for publication.

Acknowledgements: The authors would like to thank all junior doctor respondents who kindly gave their time to complete the survey. We also wish to thank members of the PPIE group who provided valuable input throughout the study.

Competing interests: None

Funding: The study was funded by NIHR Research for Patient Benefit (PB-PG-0418-20023)

Department of Health Disclaimer: The views and opinions expressed therein are those of the authors and do not necessarily reflect those of the NIHR, NHS or the Department of Health.

Ethical approval: Ethical approval was granted by the University of Birmingham and Health Research Authority (reference number: 19/HRA/6579)

Data sharing statement: This study has not received ethical approval to share confidential data with any third party other than the study research team.

For peer review only

Reporting checklist for cross sectional study.

Based on the STROBE cross sectional guidelines.

Instructions to authors

Complete this checklist by entering the page numbers from your manuscript where readers will find each of the items listed below.

Your article may not currently address all the items on the checklist. Please modify your text to include the missing information. If you are certain that an item does not apply, please write "n/a" and provide a short explanation.

Upload your completed checklist as an extra file when you submit to a journal.

In your methods section, say that you used the STROBE cross sectionalreporting guidelines, and cite them as:

von Elm E, Altman DG, Egger M, Pocock SJ, Gotzsche PC, Vandenbroucke JP. The Strengthening the Reporting of Observational Studies in Epidemiology (STROBE) Statement: guidelines for reporting observational studies.

		Reporting Item	Page Number
Title and abstract			
Title	#1a	Indicate the study's design with a commonly used term in the title or the abstract	1
Abstract	#1b	Provide in the abstract an informative and balanced summary of what was done and what was found	1
Introduction			
Background / rationale	#2	Explain the scientific background and rationale for the investigation being reported	2-4
Objectives	#3	State specific objectives, including any prespecified hypotheses	4
Methods			
Study design	#4	Present key elements of study design early in the paper	4
Setting	#5	Describe the setting, locations, and relevant dates, including periods of recruitment, exposure, follow-up, and data collection	4

For peer review only

Eligibility criteria	#6a	Give the eligibility criteria, and the sources and methods of selection of participants.	4
	#7	Clearly define all outcomes, exposures, predictors, potential confounders, and effect modifiers. Give diagnostic criteria, if applicable	4-5
Data sources / measurement	#8	For each variable of interest give sources of data and details of methods of assessment (measurement). Describe comparability of assessment methods if there is more than one group. Give information separately for for exposed and unexposed groups if applicable.	4-5
Bias	#9	Describe any efforts to address potential sources of bias	
Study size	#10	Explain how the study size was arrived at	
Quantitative variables	#11	Explain how quantitative variables were handled in the analyses. If applicable, describe which groupings were chosen, and why	5-6
Statistical methods	#12a	Describe all statistical methods, including those used to control for confounding	5-6
Statistical methods	#12b	Describe any methods used to examine subgroups and interactions	5-6
Statistical methods	#12c	Explain how missing data were addressed	5-6
Statistical methods	#12d	If applicable, describe analytical methods taking account of sampling strategy	
Statistical methods	#12e	Describe any sensitivity analyses	5-6
Results			
Participants	#13a	Report numbers of individuals at each stage of study—eg numbers potentially eligible, examined for eligibility, confirmed eligible, included in the study, completing follow-up, and analysed. Give information separately for for exposed and unexposed groups if applicable.	6
Participants	#13b	Give reasons for non-participation at each stage	N/A
Participants	#13c	Consider use of a flow diagram	N/A
Descriptive data	#14a	Give characteristics of study participants (eg demographic, clinical, social) and information on exposures and potential confounders. Give information separately for exposed and unexposed groups if applicable.	6
Descriptive data	#14b	Indicate number of participants with missing data for each variable of interest	6

For peer review only

Outcome data	#15	Report numbers of outcome events or summary measures. Give information separately for exposed and unexposed groups if applicable.	N/A
Main results	#16a	Give unadjusted estimates and, if applicable, confounder-adjusted estimates and their precision (eg, 95% confidence interval). Make clear which confounders were adjusted for and why they were included	7-10
Main results	#16b	Report category boundaries when continuous variables were categorized	7-10
Main results	#16c	If relevant, consider translating estimates of relative risk into absolute risk for a meaningful time period	
Other analyses	#17	Report other analyses done—e.g., analyses of subgroups and interactions, and sensitivity analyses	
**Discussion**			
Key results	#18	Summarise key results with reference to study objectives	10
Limitations	#19	Discuss limitations of the study, taking into account sources of potential bias or imprecision. Discuss both direction and magnitude of any potential bias.	13
Interpretation	#20	Give a cautious overall interpretation considering objectives, limitations, multiplicity of analyses, results from similar studies, and other relevant evidence.	10-13
Generalisability	#21	Discuss the generalisability (external validity) of the study results	13
**Other Information**			
Funding	#22	Give the source of funding and the role of the funders for the present study and, if applicable, for the original study on which the present article is based	14

None The STROBE checklist is distributed under the terms of the Creative Commons Attribution License CC-BY. This checklist can be completed online using https://www.goodreports.org/, a tool made by the EQUATOR Network in collaboration with Penelope.ai

For peer review only
